# Functional analysis of the dehydratase domains of the PUFA synthase from *Emiliania huxleyi* in *Escherichia coli* and *Arabidopsis thaliana*

**DOI:** 10.1186/s13068-022-02223-w

**Published:** 2022-11-15

**Authors:** Bihan Chen, Feng Wang, Xi Xie, Huifan Liu, Dongjie Liu, Lukai Ma, Gengsheng Xiao, Qin Wang

**Affiliations:** 1 Guangdong Provincial Key Laboratory of Lingnan Specialty Food Science and Technology, Zhongkai University of Agriculture and Engineering, Guangzhou, China; 2 College of Light Industry and Food, Zhongkai University of Agriculture and Engineering, Guangzhou, China

**Keywords:** Polyunsaturated fatty acid synthase, Dehydratase domain, *Emiliania huxleyi*, *Escherichia coli*, *Arabidopsis thaliana*, Recombinant, Overexpression, Complementation, Site-directed mutagenesis

## Abstract

**Background:**

Polyunsaturated fatty acid (PUFA) synthase is a multi-domain mega-enzyme that effectively synthesizes a series of PUFAs in marine microorganisms. The dehydratase (DH) domain of a PUFA synthase plays a crucial role in double bond positioning in fatty acids. Sequencing results of the coccolithophore *Emiliania huxleyi* (*E. huxleyi, Eh*) indicated that this species contains a PUFA synthase with multiple DH domains. Therefore, the current study, sought to define the functions of these DH domains (*Eh*DHs), by cloning and overexpressing the genes encoding FabA-like *Eh*DHs in *Escherichia coli* (*E. coli*) and *Arabidopsis thaliana* (*A. thaliana*).

**Results:**

A complementation test showed that the two FabA-like DH domains could restore DH function in a temperature-sensitive (*Ts*) mutant. Meanwhile, overexpression of FabA-like *Eh*DH_1_ and *Eh*DH_2_ domains increased the production of unsaturated fatty acids (UFAs) in recombinant *E. coli* by 43.5–32.9%, respectively. Site-directed mutagenesis analysis confirmed the authenticity of active-site residues in these domains. Moreover, the expression of tandem *Eh*DH_1_-DH_2_ in *A. thaliana* altered the fatty acids content, seed weight, and germination rate.

**Conclusions:**

The two FabA-like DH domains in the *E. huxleyi* PUFA synthase function as 3-hydroxyacyl-acyl carrier protein dehydratase in *E. coli*. The expression of these domains in *E. coli* and *A. thaliana* can alter the fatty acid profile in *E. coli* and increase the seed lipid content and germination rate in *A. thaliana*. Hence, introduction of DH domains controlling the dehydration process of fatty acid biosynthesis in plants might offer a new strategy to increase oil production in oilseed plants.

**Supplementary Information:**

The online version contains supplementary material available at 10.1186/s13068-022-02223-w.

## Background

Very long-chain polyunsaturated fatty acids (VLCPUFAs), such as arachidonic acid (ARA, 20: 4, n-6), eicosapentaenoic acid (EPA, 20:5, n-3), and docosahexaenoic acid (DHA, 22:6, n-3), contain more than 18 carbon atoms and more than two double bonds. VLCPUFAs play critical roles in protecting human health by maintaining the structure and function of cell membrane lipids, improving neuron development by modulating endocannabinoids in the synaptic system, promoting growth and development of rod and cone cells, and regulating physiological activities by functioning as precursors of bioactive compounds [1, 2]. These VLCPUFAs can be synthesized through both aerobic and anaerobic reaction pathways in living organisms. In the aerobic pathway common in eukaryotes VLCPUFA synthesis is catalyzed by two types of enzymes. The double bonds of PUFAs are synthesized by an oxygenic desaturation process catalyzed by different substrate-specific desaturases, and their fatty acid chains are elongated by elongases [3–5]. In the anaerobic pathway, which only occurs in prokaryotic and eukaryotic microbes [6, 7], PUFAs are synthesized by a mega-enzyme PUFA synthase [8–15].

PUFA synthase is a polyketide synthase (PKS)-like mega-enzyme that synthesizes VLCPUFAs from acetate de novo through iterative cyclic reactions [4, 16, 17]. Genome sequencing has shown that microbial PUFA synthases comprise one or more subunits, which contain more than one catalytic domain, such as ketoacyl carrier protein synthase (KS) [18], ketoacyl carrier protein reductase (KR) [19], desaturase (DH) [20], enoyl carrier protein reductase (ER) [21], malonyl-CoA: carrier protein transacylase (MAT) [22], and acyl carrier protein (ACP) [2]. In particular, two types of DH domains participate in the dehydration process for double bond introduction. The PKS-like DH domain primarily functions in the synthesis of 2-trans-enoyl-ACP, which is subsequently reduced by the ER domain to a saturated acyl-ACP, whereas the consecutive FabA-like DH domains catalyze the dehydration reaction for producing a 3-cis-enoyl-ACP, in which the 3-cis double bond is retained through subsequent condensation, catalyzed by the KS domain for VLCPUFA biosynthesis [20, 23]. However, the substrate specificity and functions of the FabA-like DH domains from distinct species remain unclear.

*Emiliania huxleyi* is one of the most abundant and widely distributed marine microalgae. This phytoplankton can accumulate several omega-3 VLCPUFAs, including docosapentaenoic acid (DPA, 22:5, n-3), EPA, and DHA, which are health-promoting nutraceutical compounds [24–27]. Recently, several elongases and desaturases of aerobic pathway from *E. huxleyi* were identified and characterized, revealing that *E. huxleyi* can synthesize VLCPUFAs through the aerobic pathway. However, certain desaturases for the biosynthesis of some PUFAs in aerobic pathway were missing inferring that an alternative pathway might participate in synthesizing these fatty acids [25, 28–30]. Genome sequencing showed that *E. huxleyi* comprises a PUFA synthase-like gene cluster with a complete component of the standard PUFA synthase, and this enzyme might involve in synthesizing PUFAs in anaerobic pathway. However, the functions of its domains have not yet been determined. This PUFA synthase-like enzyme contains two FabA-like DH domains that may process dehydratase activity for VLCPUFA biosynthesis [18, 31].

The current study sought to functionally characterize the two FabA-like DH domains from the PUFA synthase-like enzyme of *E. huxleyi.* To this work, the domains were identified by multiple sequence alignments and homology modeling. They were subsequently described in *E. coli* for their functional analysis through complementation test, site-directed mutagenesis, and overexpression. In addition, the consecutive DH domains were expressed as a heterologous enzyme in *A. thaliana* to assess the ability of the DH domains to regulate seed weight, fatty acid composition, lipid production, and germination rate. Collectively, the findings of this study provide insights regarding the functions of the *E. huxleyi* FabA-like DH domains and highlight their potential for enhanced lipid production in oilseed plants.

## Results

### Sequence analysis of the two PUFA synthase FabA-like DH domains from *E. huxleyi*

**Fig. 1:**
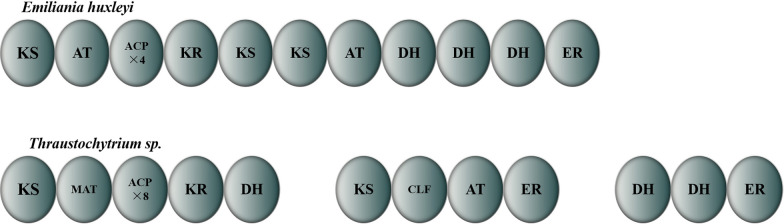
Structure of polyunsaturated fatty acid synthases from *Emiliania huxleyi* and *Thraustochytrium sp*. *ACP* acyl carrier protein, *KS* 3-ketoacyl ACP synthase, *MAT* malonyl-CoA: ACP transacylase, *KR* 3-ketoacyl-ACP reductase *DH* 3-hydroxyacyl-ACP dehydratase, *CLF* chain length factor, *AT* acyltransferase, *ER* enoyl-ACP reductase

**Fig. 2:**
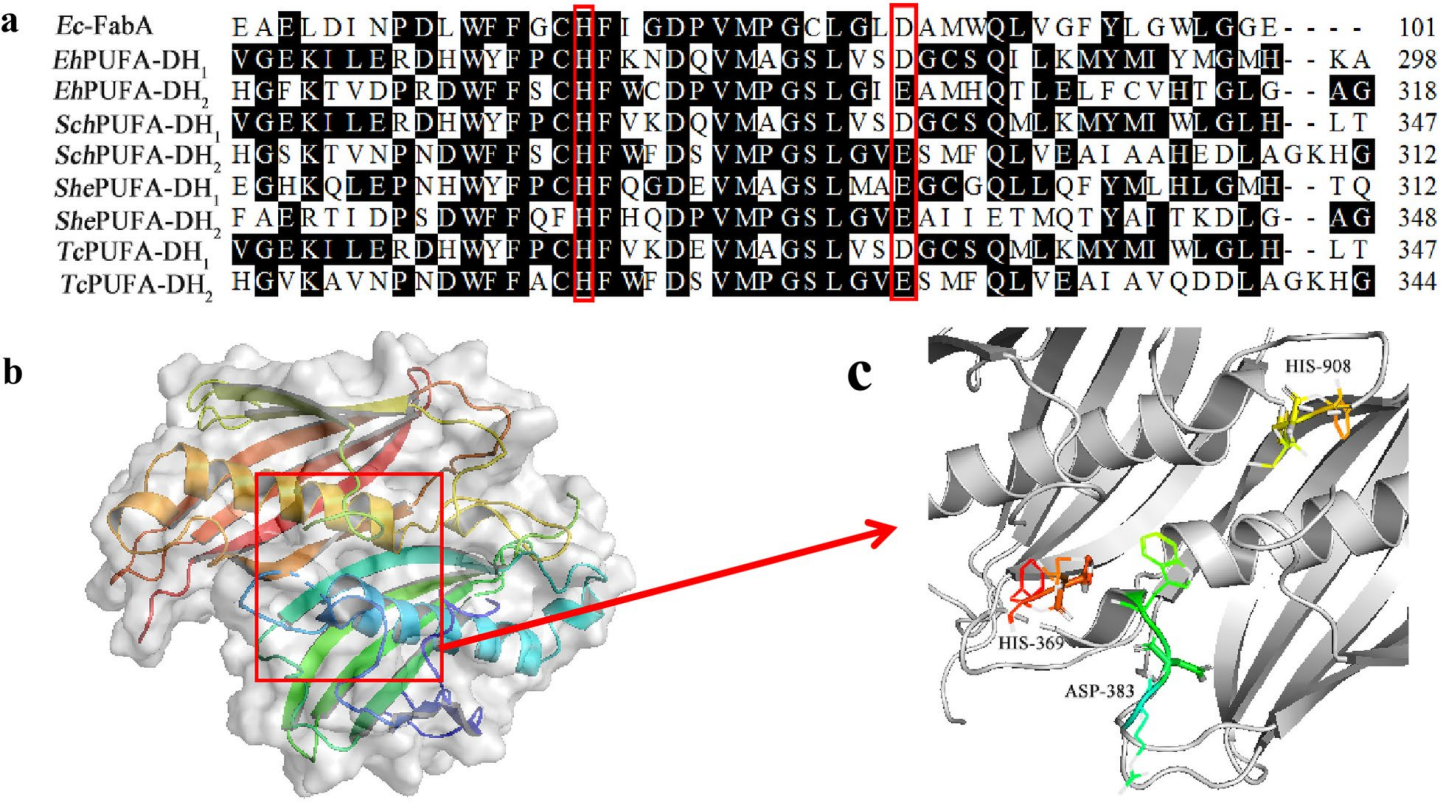
Sequence and 3D structural analysis of two FabA-like dehydratase (DH) domains from *Emiliania huxleyi* (*Eh*). **a** Multiple sequence alignment of the DH domains of PUFA synthases and FabA from *Escherichia coli* (*Ec*). **b** 3D structures of *Eh*DH_1_-DH_2_ domains. **c** Two types of predicted active-site residues on *Eh*DH_1_-DH_2_ domains. The putative active sites are highlighted by a red rectangle, and the consensus amino acids are shaded. *Ec*FabA, FabA from *E.coli*; *Eh*PUFA-DH_1_, DH_1_ domain of polyunsaturated fatty acid (PUFA) synthase from *Emiliania huxleyi*; *Eh*PUFA-DH_2_, DH_2_ domain of PUFA synthase from *Emiliania huxleyi*; *Sch*PUFA-DH_1_, DH_1_ domain of PUFA synthase from *Schizochytrium*; *Sch*PUFA-DH_2_, DH_2_ domain of PUFA synthase from *Schizochytrium*; *She*PUFA-DH_1_, DH_1_ domain of PUFA synthase from *Shewanella*; *She*PUFA-DH_2_, DH_2_ domain of PUFA synthase from *Shewanella*; *Tc*PUFA-DH_1_, DH_1_ domain of PUFA synthase from *Thraustochytrium*; *Tc*PUFA-DH_2_, DH_2_ domain of PUFA synthase from *Thraustochytrium*

The genome sequencing (GenBank: XP_005774327.1) results showed that the PUFA synthase-like mega-enzyme from *E. huxleyi* comprises multiple catalytic domains (Fig. 1), including one PKS-like DH and two tandem FabA-like DH domains (*Eh*DH_1_ and *Eh*DH_2_) at the C-terminus of the mega-enzyme [24]. As the FabA-like DH domains of *Thraustochytrium* sp. 26,185 PUFA synthase (named *Tc*DH domains) have been characterized in our previous studies [32, 36], the *Tc*DH domains were used as the reference domains for functional analysis of DH domains of PUFA synthase from *E. huxleyi.* The multiple sequence alignment further revealed that the two FabA-like *Eh*DH domains shared amino acid identities of 24.0–25.7% with the *E. coli* FabA domains and 44.0–36.9% with the FabA-like DH domains from the *Thraustochytrium* sp. 26,185 PUFA synthase, respectively. To obtain additional structural information, we employed PyMOL software (https://pymol.org/2/) to construct three-dimensional (3D) models of the two domains. As a result, the tandem *Eh*DH_1_-DH_2_ domain was found to be similar to 3-hydroxydecanoyl-ACP dehydratase (FabA), particularly that of *E. coli*, *Pseudomonas aeruginosa*, *Vibrio cholerae*, and *Yersinia pestis*. FabA (PDB ID: 951,844) was then applied as a template to build the *Eh*DH_1_-DH_2_ domains. The sequence identity of the model was 34.53%. According to the Ramachandran plot, approximately 90% of amino acids were within the most favored regions, while no amino acids were in the disallowed region, confirming that the model was reasonable and suitable for follow-up studies. The Errata overall quality factor score of the model was 87.41. Theoretically, a score greater than 85 is considered good, indicating that the model meets the desired requirements. The Verify 3 D results showed that 94.86% of the residues had averaged 3 D-1 D scores  ≥ 0.2, with more than 80% of qualified lines indicating good model reliability. The homology modeling of *Eh*DH_1_ and *Eh*DH_2_ domains revealed the presence of a long pseudo-domain sequence and a core FabA-like DH domain [32]. Two predicted active-site residues, histidine (H) and aspartic acid/glutamic acid (D/E), were found in the middle of each core domain [18] (Fig. 2a). The structure analysis revealed that the pseudo-domain and core domain had similar “hot-dog” structures with a core helix surrounded by two β-sheets. These domains were connected forming a double “hot-dog” structure (Fig. 2b). For the core domain, the conserved catalytic H residue was within the loop region in the initial region of the central hot-dog helix, while the conserved D/E residue resided in the hot-dog helix [33]. However, the upstream long pseudo-domain lacked one active-site residue (D) in the sequence, with the remaining active-site residues reacted with the those of the core DH domain to form a “D–H–H” catalytic triad. Therefore, the predicted active-site residue H was identified at positions 369 and 908 of *Eh*DH_1_-DH_2_, while another active-site residue from the pseudo-domain was identified at position 383 of the domain (Fig. 2c).

### Heterologous complementation of *E. coli* FabA mutant with DH domains

FabA acts as the key enzyme for UFA biosynthesis in *E. coli* by catalyzing the dehydration of 3-hydroxyacyl-ACP to a cis-3-enoyl-ACP with a 10-carbon substrate [34]. The product cis-3-enoyl-ACP was further condensed by a β-ketoacyl-acyl carrier protein synthase I (FabB), resulting in the cis-double bond being retained on UFAs. The lack of FabA function leads to loss of UFAs, and subsequent dysregulation of normal cell membrane function and inhibition of cell growth [35].

**Fig. 3:**
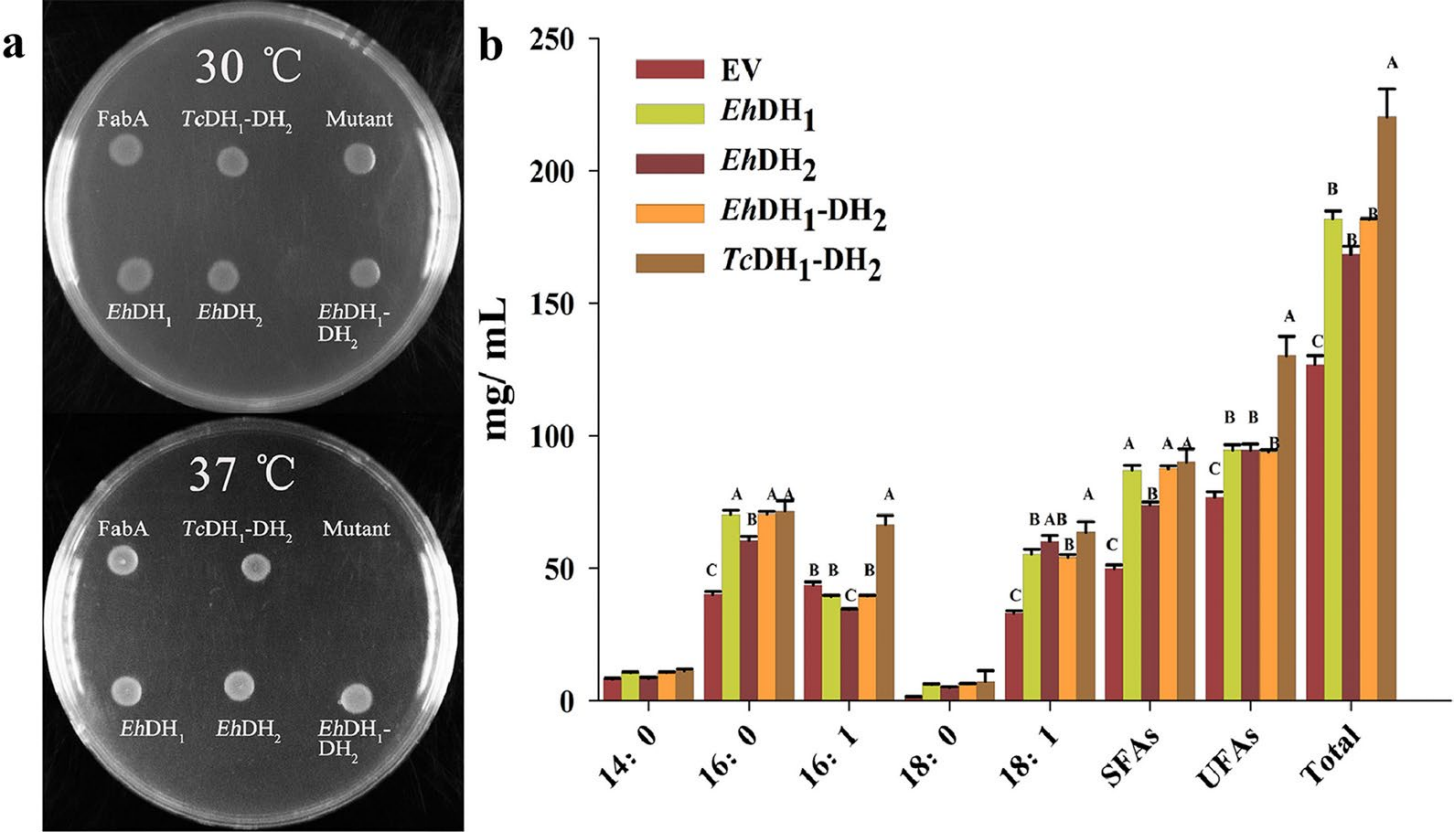
Functional analysis of *E*hDH domains in *Escherichia coli (E. coli)*. **a** Complementation plate assay of the dehydratase (DH) domains in *E. coli* FabA Temperature-sensitive (*Ts*) mutant. DH domains were individually inserted behind the inducing promoter pBAD. The transformants grown on 0.02% L-arabinose selection plates were incubated overnight at 30 and 37 ℃, respectively. FabA, *E. coli* wild type dehydratase; *Tc*DH_1_-DH_2_, the mutant expresses tandem DH_1_-DH_2_ domains from *Thraustochytrium*; Mutant, *E. coli* FabA *Ts* mutant with an empty vector; *Eh*DH_1_, the mutant expresses the DH_1_ domains from *Emiliania huxleyi*; *Eh*DH_2_, the mutant expresses the *Eh*DH_2_ domains; *Eh*DH_1_-DH_2_, the mutant expresses both *Eh*DH_1_ and *Eh*DH_2._ **b** The amount of fatty acid production in *E. coli* expressing the DH domains. Fatty acid production in *E. coli* Rosetta strains overexpressing DH domains. EV, *E. coli* Rosetta with an empty vector; *Eh*DH_1_, *E. coli* Rosetta overexpressing *Eh*DH_1_ domain; *Eh*DH_2_, *E. coli* Rosetta overexpressing *Eh*DH_2_ domain; *Eh*DH_1_-DH_2_, *E. coli* Rosetta overexpressing both *Eh*DH_1_ and *Eh*DH_2_; *Tc*DH_1_-DH_2_, *E. coli* Rosetta overexpressing both *Tc*DH_1_ and *Tc*DH_2._ Fatty acid abbreviations are the following: 14:0, myristic acid; 16:0, palmitic acid; 16:1, palmitoleic acid; 18:0, octadecanoic acid; 18:1, cis-vaccenic acid; *SFAs* saturated fatty acids, *UFAs* unsaturated fatty acids, *Total* total fatty acids. Values are reported as the means ± standard deviation of three independent biological replicates. Means with the same letters do not differ significantly. Statistical analysis of the results was conducted using one-way analysis of variance (*P* < 0.05)

To perform functional characterization of the DH domain, the DH domain complementation test was carried out in a FabA (*Ts*) mutant with a temperature-sensitive phenotype. FabA (*Ts*) normally contains mutations in the *FabA* gene that enable the mutant to grow at lower temperatures (30 °C), while inhibiting growth at nonpermissive temperatures (37 °C). As shown in Fig. 3a, the mutants expressing two FabA-like *Eh*DH domains from the PUFA synthase of *E. huxleyi*, tandem FabA-like DH domains from the PUFA synthase of *Thraustochytrium* sp. 26,185 (*Tc*DH_1_-DH_2_), and wild-type FabA were all capable of growing at both temperatures, while the mutant containing an empty vector ceased growth at 37 °C. These findings indicate that *Eh*DH_1_ and *Eh*DH_2_ domains can restore the 3-hydroxyacyl-ACP DH activity in *E. coli* by synthesizing UFAs.

To further define the *Eh*DH domain functions, the genes encoding *Eh*DH_1_, *Eh*DH_2_, *Eh*DH_1_-DH_2_, and *Tc*DH_1_-DH_2_ were inserted into the plasmid vector pET32a. They were overexpressed under a strong promoter in the wild-type *E. coli Rosetta* strain. After inducible expression for 12 h, the total fatty acids of the recombinant strains with different individual DH domains were extracted. The production of five major fatty acids, namely, myristic acid (C14:0), palmitic acid (C16:0), palmitoleic acid (C16:1–9), stearic acid (18:0), and oleic acid (C18:1–11), was analyzed. Overexpressions of all DH domains from the PUFA synthase lead to markedly higher levels of total fatty acids compared to the empty vector control (Fig. 3b). In particular, the recombinants comprising *Eh*DH_1_, *Eh*DH_2_, and *Eh*DH_1_-DH_2_ produced significantly higher amounts of saturated fatty acids (SFAs) and UFAs, such as C16:0, C18:0, and C18:1, compared with the empty vector control. The recombinants containing *Eh*DH_1_ and *Eh*DH_1_-DH_2_ domains accumulated similar levels of fatty acids, but more SFAs, than the recombinants expressing *Eh*DH_2_. The total fatty acid production of the recombinants with two tandem DH domains was 181.90 mg/L, showing an increase of approximately 43.50% over the control. When the UFA/SFA ratios of these strains were compared, all recombinants overexpressing DH domains produced lower UFA/SFA ratios than the empty vector control, which shared a similar result with the overexpression of the *FabA* gene in *E. coli* and overexpression of the *DH domain* gene in *Thraustochytrium* sp. 28,165 (*Tc*) (Additional file 1: Table S1) [36].

### Site-directed mutagenesis analysis of the DH domains

**Fig. 4:**
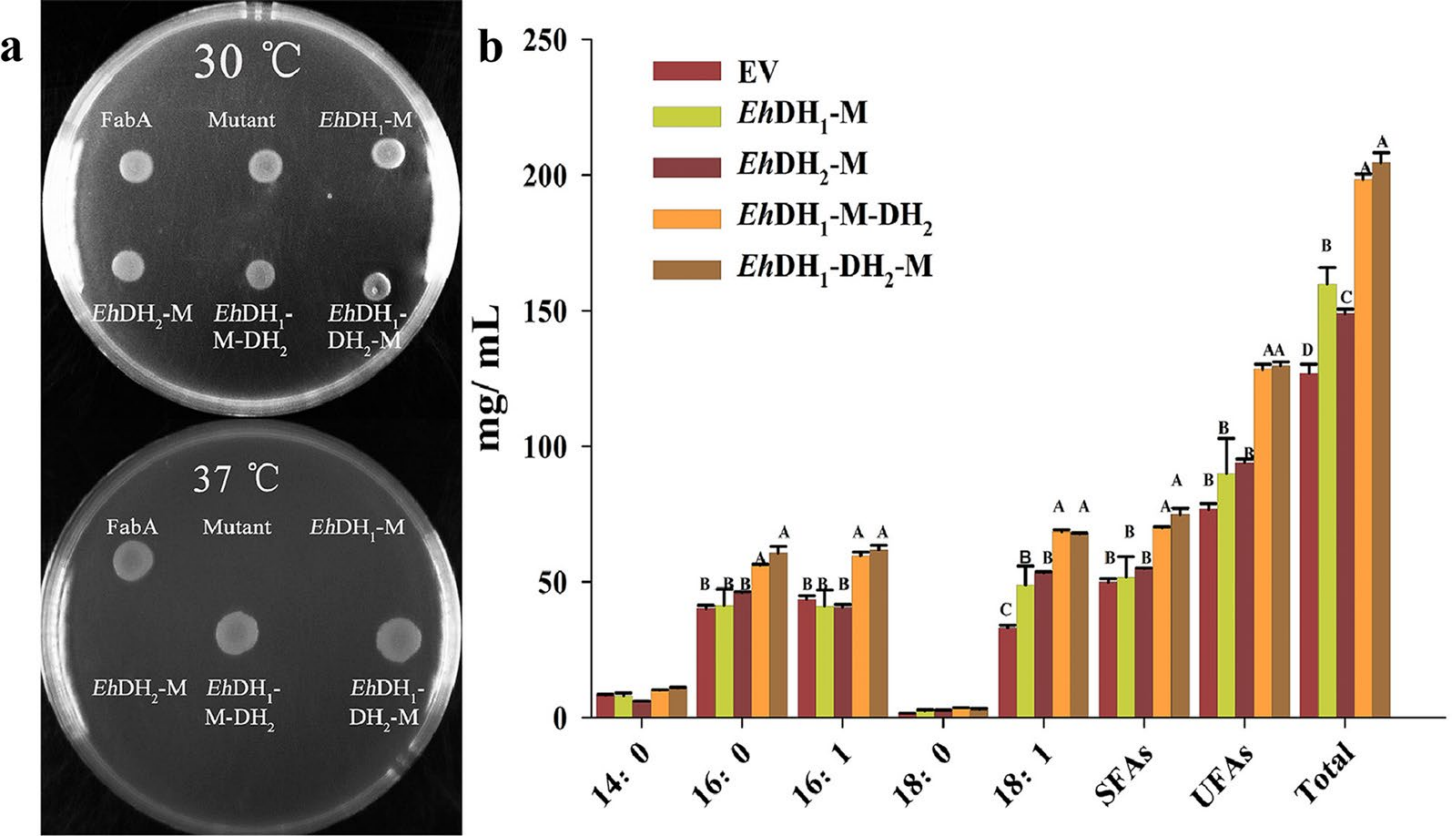
Functional validation of site-directed mutagenesis of *Eh*DH domains. **a** Complementation plate assay of the dehydratase (DH) domains in *Escherichia coli* (*E. coli*) FabA Temperature-sensitive (*Ts*) mutant as conducted in Fig. 3. FabA, *E. coli* wild type dehydratase; Mutant, *E. coli* FabA *Ts* mutant with an empty vector; *Eh*DH_1_-M, the mutant expresses the mutated DH_1_ domains from *Emiliania huxleyi*; *Eh*DH_2_-M, the mutant expresses the mutated *Eh*DH_2_ domains; *Eh*DH_1_-M-DH_2_, the mutant expresses the mutated DH_1_ along with DH_2_; *Eh*DH_1_-DH_2_-M, the mutant expresses the mutated DH_2_ along with DH_1_. **b** Fatty acid production following DH overexpression in wild-type *E. coli*. EV, *E. coli* Rosetta with an empty vector; *Eh*DH_1_-M, *E. coli* Rosetta overexpressing the mutated *Eh*DH_1_ domain; *Eh*DH_2_-M, *E. coli* Rosetta overexpressing the mutated *Eh*DH_2_ domain; *Eh*DH_1_-M-DH_2_, *E. coli* Rosetta overexpressing the mutated DH_1_ along with DH_2_; *Eh*DH_1_-DH_2_-M, *E. coli* Rosetta overexpressing the mutated DH_2_ along with DH_1_. Fatty acid abbreviations are the following: 14:0, myristic acid; 16:0, palmitic acid; 16:1, palmitoleic acid; 18:0, octadecanoic acid; 18:1, cis-vaccenic acid; *SFAs* saturated fatty acids, *UFAs* unsaturated fatty acids, *Total* total fatty acids. Values are reported as the means ± standard deviation of three independent biological replicates. Means with the same letters do not differ significantly. Statistical analysis of the results was conducted using one-way analysis of variance (*P* < 0.05)

According to the multiple amino acid sequence alignment, the conserved catalytic active-site residues of the *Eh*DH_1_ and *Eh*DH_2_ domains were inactivated to confirm the authenticity of the functional DH domains. Additionally, the active-site D or E residues were substituted by alanine (A) residues through site-directed mutagenesis. The results of the sodium dodecyl sulphate-polyacrylamide gel electrophoresis (SDS-PAGE) and western blotting (WB) analyses confirmed the expression of all cloned *Eh*DH genes (Additional file 1: Fig. S1). The mutated *Eh*DH_1_, *Eh*DH_2_, and *Eh*DH_1_-DH_2_ domains were expressed as stand-alone enzymes in the FabA (*Ts*) mutant for the complementation analysis. Similar to the empty vector control, the recombinant overexpressing mutated domains could grow at 30 °C but not at 37 °C (Fig. 4a), revealing that the inserted mutated *Eh*DH fragments were non-functional in the mutants. In contrast, the recombinants overexpressing wild-type *Eh*DH domains grew at both permissive and nonpermissive temperatures. These experiments confirmed that the putative catalytic active site was crucial for the domain function.

In addition, these mutant domains were overexpressed in the wild-type *E. coil Rosetta* strain (Fig. 4b). The recombinant overexpressing mutated DH domains accumulated similar levels of fatty acids as the empty vector control, while the recombinants overexpressing at least one wild-type DH domain produced higher levels of SFAs and UFAs. Moreover, the expression of the two tandem *Eh*DH domains, with either mutated, revealed a slightly stronger effect of the mutated *Eh*DH_1_ (*Eh*DH_1_-M-DH_2_) over the mutated *Eh*DH_2_ (DH_1_-DH_2_-M).

### Generation of the recombinant construct expressing tandem *Eh*DH_1_-DH_2_ domains in *A. thaliana*

Considering that the tandem DH domains could regulate fatty acid production in *E. coli*, these domains were further functionally analyzed by strong promoter-driven overexpression in the model oilseed plant *A. thaliana*. The binary plasmid comprising three expression cassettes was constructed to express the tandem *Eh*DH domains in *A. thaliana.* The first cassette was used to express the tandem *Eh*DH domains as a stand-alone protein with a chloroplast transit peptide from *A. thaliana* fused to the N-terminus under the control of the 35S promoter. The second cassette was used to express a kanamycin-resistance gene under the control of a constitutive promoter for selecting transgenic plants. The third cassette was used to express a modified green fluorescent protein (eGFP) from *Aequorea victoria* under the 35S promoter for screening transgenic seeds (Additional file 1: Fig. S2).

### Expression of the *Eh*DH_1_-DH_2_ domain in wild-type *A. thaliana*

**Fig. 5:**
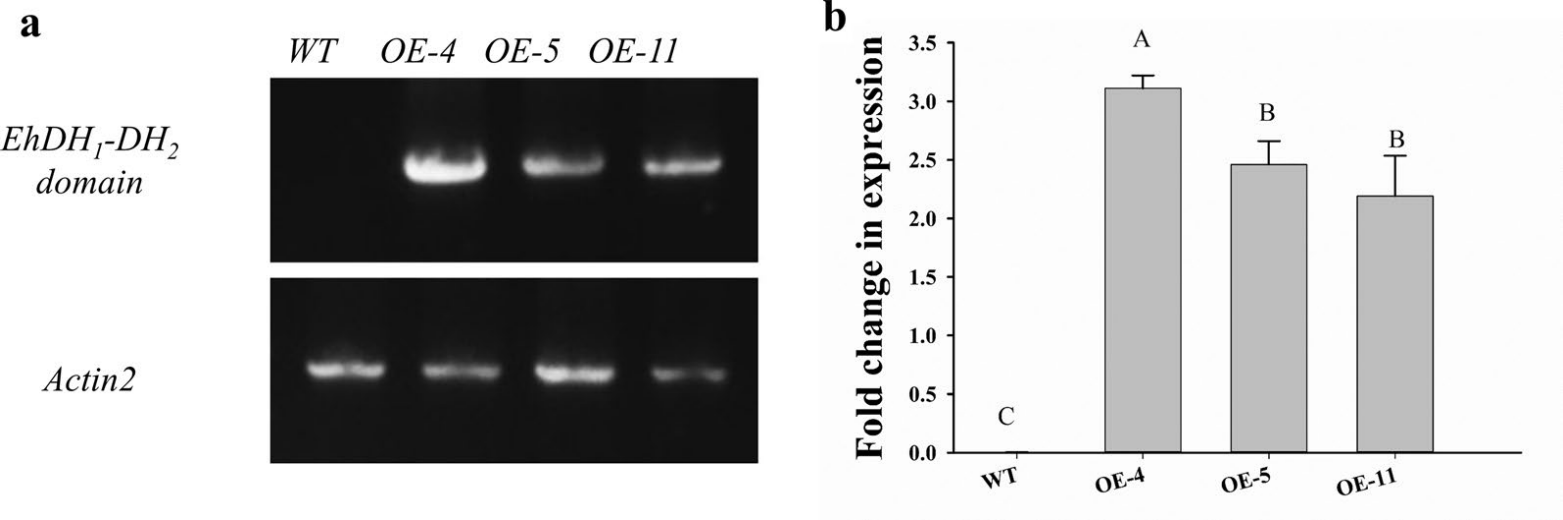
Transcriptional analysis of the *EhDH*_*1*_*-DH*_*2*_ domain in developing siliques of overexpression lines. **a** Expression of the *Eh*DH_1_-DH_2_ domain and *AtActin 2* in the developing siliques of *EhDH1-DH2/WT* analyzed by semi-quantitative RT-PCR. **b** Expression of the *EhDH*_*1*_*-DH*_*2*_ domain in developing siliques of *EhDH*_*1*_*-DH*_*2*_*/WT* analyzed by quantitative RT-PCR. *WT* wild type, *OE* overexpression lines. Values are reported as the means–standard deviation of three independent biological replicates. Means with the same letters do not differ significantly. Statistical analysis of the results was conducted using one-way analysis of variance (*P* < 0.05)

To assess the effect of the *Eh*DH_1_-DH_2_ domain in fatty acid biosynthesis and oil production in *A. thaliana*, the construct expressing the *Eh*DH_1_-DH_2_ domain under the 35S promoter was introduced into wild-type *A. thaliana*. Transgenic seeds were selected by the fluorescence signal and propagated to the next generation. To confirm the expression of the *Eh*DH_1_-DH_2_ domain, total RNAs were isolated from the developing siliques of three transgenic lines (OE-4, OE-5, OE-11) and reverse-transcribed to cDNAs (Fig. 5b, a). Quantitative PCR analysis of the cDNAs showed that the transcription levels of the *Eh*DH_1_-DH_2_ domain were highly varied among the three overexpression (OE) lines, with the highest levels observed in OE-4, followed by OE-5 and OE-11 (Fig. 5b).

### Expression and early seedling growth of the *Eh*DH_1_-DH_2_ domain in OE lines of *A. thaliana*

**Fig. 6:**
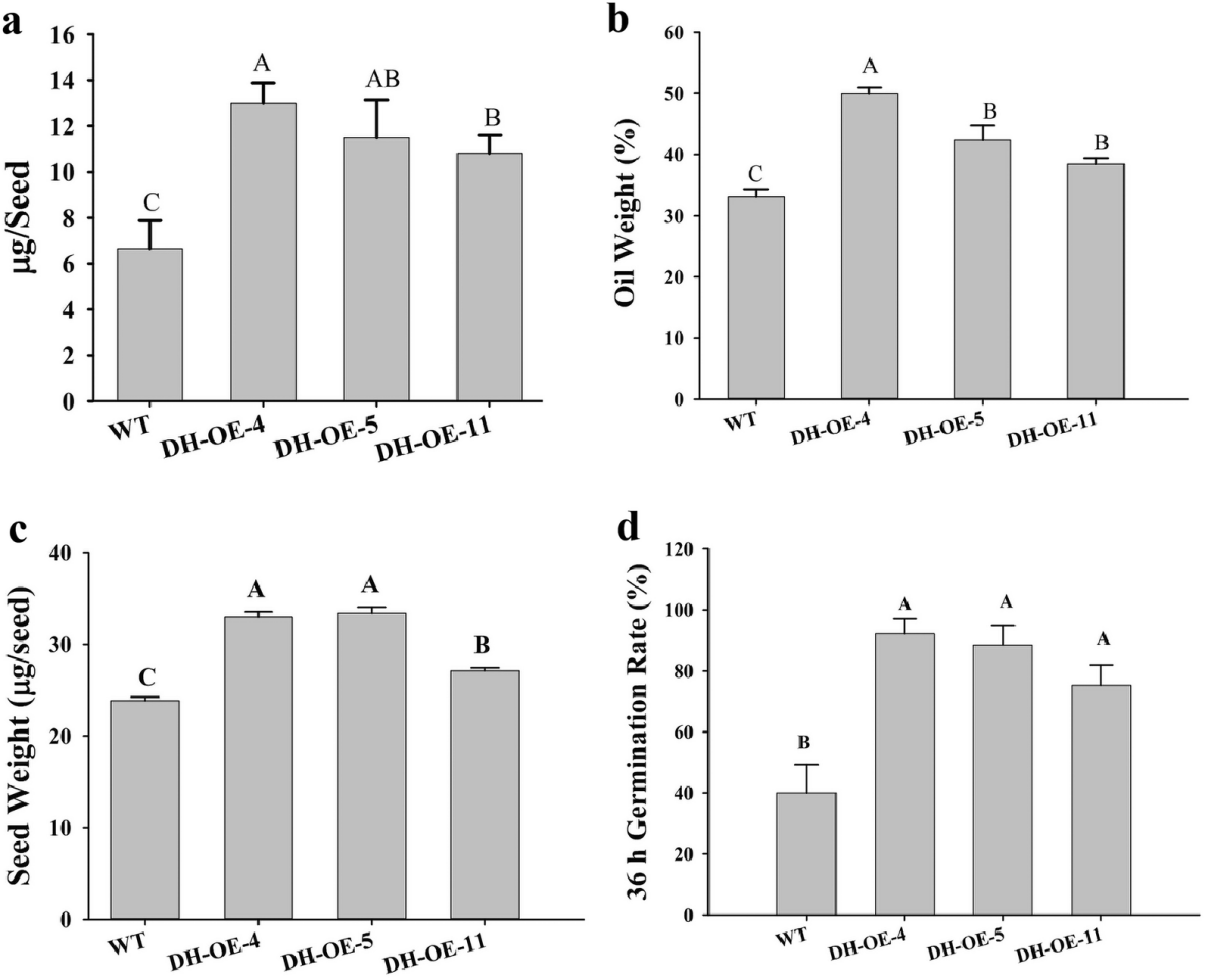
Effect of the expression of tandem *Eh*DH domains in *Arabidopsis.* **a** Amount of oil per seed in the T2 seeds of overexpression lines. **b** Seed oil content of the transgenic lines. **c** Seed weight of the transgenic lines. **d** Germination rates of wild-type and transgenic lines at 36 h of imbibition. *WT* wild type, *OE* overexpression lines. Values are reported as the means ± standard deviation of 10 independent biological replicates. Means with the same letters do not differ significantly. Statistical analysis of the results was conducted using one-way analysis of variance (*P* < 0.05)

To examine the effect of the *Eh*DH_1_-DH_2_ domain in fatty acid biosynthesis and oil accumulation in *A. thaliana*, the seed oil content and mass of these three transgenic lines were measured. All transgenic lines accumulated more oil relative to the wild-type control (Fig. 6). The amount of oil per seed in OE-4, OE-5, OE-11 increased by 95.80%, 73.20%, and 62.70%, respectively, compared with the control (Fig. 6a). In particular, the levels of monounsaturated fatty acids, such as C18:1 and C20:1, were significantly higher in these transgenic lines than in the wild-type control [37]. Moreover, the transgenic lines exhibited a decrease in total SFAs and increase in total UFAs; however, their UFA/SFA ratios were 2.61, 2.50, and 2.50 times that of the negative control, respectively (Table 1). The percentages of oil content in these transgenic lines increased by 50.80%, 28.10% and 13.90%, respectively, compared with the control (Fig. 6b). Moreover, the transgenic seeds were significantly heavier, representing 1.38-, 1.36-, and 1.14-fold increase, respectively, relative to the wild-type seeds (Fig. 6c). These results indicated that expressing the tandem *Eh*DH domains can significantly increase seed oil content and seed mass in *A. thaliana.*

**Table 1:** Fatty acid composition (mol%) of transgenic seeds overexpressing tandem *Eh*DH domains

FA species	WT	DH-T2-4	DH-T2-5	DH-T2-11
16:0	12.05 ± 0.83^A^	7.66 ± 0.31^C^	7.33 ± 0.27^C^	8.28 ± 0.31^B^
16:1–9	1.66 ± 0.20^A^	0.33 ± 0.03^B^	0.54 ± 0.15^B^	0.37 ± 0.01^B^
18:0	10.73 ± 1.32^A^	4.26 ± 0.28^B^	4.59 ± 0.37^B^	4.33 ± 0.24^B^
18:1–9	11.19 ± 0.82^C^	15.67 ± 0.49^A^	14.46 ± 0.30^B^	14.6 ± 0.44^B^
18:1–11	2.26 ± 0.23^A^	1.3 ± 0.08^B^	1.24 ± 0.05^B^	1.33 ± 0.03^B^
18:2	20.81 ± 0.74^C^	24.95 ± 0.44^AB^	25.54 ± 0.35^A^	24.01 ± 0.53^B^
18:3	14.19 ± 1.23^C^	16.78 ± 0.59^B^	17.1 ± 0.37^B^	18.63 ± 0.46^A^
20:1–11	14.2 ± 0.54^B^	20.93 ± 0.14^A^	20.4 ± 0.47^A^	20.54 ± 0.70^A^
Others	12.91 ± 1.12	8.12 ± 0.15	8.8 ± 0.21	7.92 ± 0.14
C16–18	72.89 ± 0.77	70.95 ± 0.22	70.80 ± 0.63	71.54 ± 0.73
SFA	32.06 ± 1.21^A^	15.31 ± 0.65^B^	15.84 ± 0.82^B^	15.90 ± 0.69^B^
UFA	67.94 ± 1.73^B^	84.69 ± 0.65^A^	84.16 ± 0.82^A^	84.10 ± 0.69^A^
UFA/SFA	2.12	5.53	5.31	5.29

*FA* fatty acid, *WT* wild type, *OE* overexpression lines, *SFA* saturated fatty acid, *UFA* unsaturated fatty acid, *UFA/SFA* ratio of unsaturated/saturated fatty acids.

Values are reported as the means ± standard deviation of 10 independent biological replicates. The means with the same letters are not statistically significantly different. The statistical analysis of the results was conducted using one-way analysis of variance (*P* < 0.05)

To examine the growth effects of the tandem *Eh*DH domains, the growth phenotypes of the transgenic *A. thaliana* plants were carefully observed during their life cycle. The 36-h germination rate in OE-4, OE-5 and OE-11 increased by 130.50%, 121.50%, and 88.30%, respectively, compared with the control (Fig. 6d). Hence, expression of the tandem *Eh*DH_1_-DH_2_ domains from the PUFA synthase in *A. thaliana* could promote seed germination.

## Discussion

VLCPUFAs, such as EPA, DPA, and DHA, are found in most living organisms and synthesized through aerobic or anaerobic pathways. The aerobic pathway widely exists in many species, synthesizing VLCPUFAs by the combined action of elongases and desaturases. However, the anaerobic pathway is an alternative pathway employed by many marine microbes [38]. In the anaerobic pathway, PUFA synthase is the key mega-enzyme that synthesizes a number VLCPUFAs de novo [17]. Sequence analysis, in the current study, showed that PUFA synthases comprise one or more subunits with multiple catalytic domains that cooperate to form VLCPUFAs with multiple double bonds. Domain studies of PUFA synthases indicated that two key domains, KS and DH, play a key role in double bond positioning in VLCPUFAs: the KS domain catalyzes the condensation of enoyl-ACP with malonyl-ACP for double bond retention, while the DH domain catalyzes the introduction of a double bond by removing a water molecule from hydroxyl-ACP, forming a cis-enoyl-ACP [39, 40]. Structural analysis of the DH domains of the PUFA synthases revealed that they possess a double “hot-dog” architecture, similar to the DH domain of the fatty acid synthase. Based on their amino acid sequences, DH domains were classified into two categories: PKS-like and FabA-like DH domains. The PKS-like DH domain catalyzes the dehydration of acyl-chain by introducing a 2-trans double bond, whereas the two FabA-like DH domains in a tandem arrangement that more closely resembles the *E. coli* dehydratase (FabA), which can introduce cis-double bonds [32, 41].

To define the functions of these PUFA synthase FabA-like DH domains, many researchers have attempted to dissect the DH domain from the PUFA synthase and investigate their activity in different expression systems. For instance, Oyola-Roble cloned two FabA-like DH domains derived from the *Photobacterium profundum* PUFA synthase and overexpressed them in *E. coli*. They found that DH domain overexpression led to accumulation of over five times the total fatty acid content compared with the wild-type control, indicating that the heterologous DH domain could regulate fatty acid production in *E. coli* [41]. Moreover, Xiao Qiu’s team analyzed the two distinct FabA-like DH domains of the PUFAs synthase of *Thraustochytrium* sp. ATCC 26,185 in *E. coli*. The complementation assay results showed that both FabA-like DH domains functioned as 3-hydroxyacyl-ACP dehydratases for the biosynthesis of UFAs in *E. coli*. Overexpression of the DH_1_ domain led to accumulation of more total fatty acids, while that of the DH2 domain yielded more UFAs; their co-expression produced high levels of total fatty acids. Subsequently, these two DH domains from the PUFAs synthase of *Thraustochytrium* sp. ATCC 26,185 were studied as a whole by domain swapping, domain complementation, and overexpression. The domain complementation test demonstrated that the DH_1_ domain could complement the DH domain defect of the fatty acid synthase in *Saccharomyces cerevisiae*. Overexpression of the DH_1_-mutated PUFA synthase failed to produce DHA in *E. coli*, while overexpression of a PUFA synthase with a mutated DH_2_ domain effectively produced DHA. In addition, Hayashi compared the substrate specificities of two purified FabA-like DH domains from PUFA synthases that could produce ω-3 and ω-6 VLCPUFAs and found that the biosynthesis of ω-3 and ω-6 VLCPUFAs is defined in the carbon length in the dehydration reaction of 3-hydroxyacyl-ACP substrates [42]. Therefore, characterizing the activity and substrate specificity of different FabA-like DH domains could elucidate the potential functions of PUFA synthases and reveal the mechanism of double bonds positioning in VLCPUFAs.

*Emiliania huxleyi,* is a coccolithophorid microalgal species that can synthesize a number of VLCPUFAs as storage lipids [27]. Genome sequencing revealed that this species might utilize two pathways for VLCPUFAs biosynthesis. For example, Δ 4, 5, 8, and 15-desaturases, as well as Δ 5, 9-elongases have been identified and characterized in the biosynthesis of a number of VLCPUFAs, including ALA, eicosatetraenoic acid (SDA, 18:4n-3), and DHA [26, 29]. Interestingly, genome sequencing revealed the presence of a PUFA synthase-like gene cluster containing all elements of a standard PUFA synthase in this species [43, 44]. This enzyme contains multiple catalytic domains, including the KS, DH, KR, AT, and ER domains, which may participate in VLCPUFA biosynthesis. To date, many research teams have endeavored to functionally characterize this PUFA synthase-like gene. However, owing to its large size encoding more than 10,000 amino acids cloning and expressing this gene has proven difficult, thus, preventing its characterization in a model expression system. Alternatively, the potential function of this enzyme could be partially characterized via analysis of its catalytic domains. In the current study, the key FabA-like DH domain was analyzed by subjecting the PUFA synthase-like protein of *E. huxleyi* (*Eh*PUFA) to sequence analysis. Two FabA-like DH domains were identified from *Eh*PUFA*.* The *Eh*DH domains share about 40% sequence similarity and conserved catalytic sites with DH domain of *Thraustochytrium* sp. 26,185 PUFA synthase (Fig. 2a). The 3D-modeling of the structure of these domains revealed that each FabA-like *Eh*DH domain possesses a double “hot-dog” structure with putative active sites, indicating that these domains might catalyze the dehydration of acyl-ACPs during VLCPUFA biosynthesis.

Given that we previously successfully characterized the dissected DH domains from a functional *Thraustochytrium* sp. 26,185 PUFA synthase via complementation testing, and overexpression in *E. coli*, the putative function of DH domains from *E. huxleyi* were characterized, herein, in a similar way. In this study, the genes encoding the FabA-like *Eh*DH domains were cloned and expressed as stand-alone enzymes in *E. coli* and *A. thaliana* to investigate their catalytic activity; the DH domain gene of *Thraustochytrium* sp. 26,185 PUFA synthase was used as a reference. Based on the method from the previous study, the FabA *Ts* mutant with low DH activity was used for the plate complementation test. This mutant cannot grow at nonpermissive temperatures due to the mutated FabA being unable to form the appropriate dimer structure for synthesizing UFAs [35, 45]. In contrast, the mutant can grow normally when it contains a functionally similar dehydratase. In the complementation test, *Eh*DH_1_ and *Eh*DH_2_ exhibited relatively low sequence similarity to *E. coli* FabA, however, both were functionally complementary to the FabA *Ts* mutants. It was speculated that these domains could restore FabA activity in *E. coli* for the biosynthesis of UFAs, indicating that the FabA-like DH domains of *E. huxleyi* were functionally similar to FabA, with analogous structures. In addition, overexpression of these domains in wild-type *E. coli* altered fatty acid production (Fig. 3). Similar to the results obtain via overexpressing the DH domain of the *Thraustochytrium* (*Tc*DH) PUFA synthase [36], expressing *Eh*DH_1_, *Eh*DH_2_, and *Eh*DH_1_-DH_2_ led to higher accumulation of UFAs and SFAs and a lower UFA/SFA ratio relative to the control (Additional file 1: Table S1). This result suggested that either tandem or individual DH domains are functionally (not only structurally) more similar to the FabA-like DH domain. Subsequently, site-directed mutagenesis was performed to examine the authenticity of the putative catalytic site from the FabA-like *Eh*DH domains. The D or E residues at the predicted active sites of these domains are highly conserved in dehydratases [46, 47]. After substituting the D or E residue to A, all substitutions failed to complement the defective *FabA Ts* mutant phenotype, confirming that the function of this amino acid, which was defined by multiple sequence alignment, is essential to the catalytic DH domains.

In plants, the biosynthesis of long-chain fatty acids occurs in plastids and is catalyzed by a type II fatty acid synthase complex with several discrete enzymes [48–50]. Similar to *E. coli*, discrete DHs participate in catalyzing the dehydration reaction and introducing a double bond in acyl-ACP for fatty acid production. As the sequential reactions are regulated by distinct enzymes, they can be impacted by the heterologous enzymes from other species [51]. Indeed, the expression of heterologous genes involved in fatty acid biosynthesis can, reportedly, alter the fatty acid levels in plants [18, 52]. In the present study, to further examine the impact of the DH domains in fatty acid biosynthesis and oil accumulation in *A. thaliana*, we overexpressed the tandem *Eh*DH_1_-DH_2_ domains in wild-type *A. thaliana*. The three transgenic lines expressing the FabA-like DH domains from *E. huxleyi* accumulated more fatty acids in their seeds, which had a higher weight, UFA content, and germination rate compared with wild-type seeds. However, the cause of the difference between the OE and control lines remains unclear. The tandem DH domains from the PUFA synthase might simply possess a higher dehydration activity. In addition, expression of the tandem DH domains of the microbial PUFA synthase in a plant might minimize possible feedback inhibition over endogenous expression [18]. Nevertheless, the UFAs to SFAs ratio differed between *E. coli* and *A. thaliana* overexpressing *Eh*DH1-DH2 domains. This phenomenon might be influenced by the different mechanism of UFAs biosynthesis between these two species. That is, in *E. coli*, the UFAs are synthesized from acetate through the iterative reactions of fatty acid synthase, the double bonds are introduced by the FabA or the FabA-like protein giving enoyl-ACPs, and the double bonds are retained by a FabB for UFA biosynthesis. The high DH expression can lead to accumulation of enoyl-ACPs, however, the FabB level is insufficient to retain all double bonds, therefore, the enoyl-ACP would be sequentially reduced by FabI and produce SFAs, resulting in a reduced UFA ratio. This phenomenon also occurs in *E. coli* overexpressing FabA [53]. Meanwhile, in plants, UFAs are synthesized from a long-chain fatty acid and the double bonds are introduced by desaturases. The overexpression of *Eh*DH1-DH2 domains can lead to accumulation of more fatty acids; the SFAs can then serve as substrates for desaturases in UFA biosynthesis. Therefore, the overexpression of fatty acid synthase gene components in plants not only increases the level of total fatty acids but also the UFA ratio [54].

Genetic modification strategies are widely used in transgenic microorganisms and plants to enhance the production of certain products through overexpression or mutation of genes involved in synthetic pathways and through knocking out genes involved in competing pathways. Currently, the most convenient way to verify enzyme function is to perform complementation experiments. In the current study, *E. coli* was selected as the model organism due to its enzyme-deficient nature and ability to synthesize double bonds. We first verified that the two FabA-like DH genes derived from *E. huxleyi* improved the PUFA-producing ability of *E. coli*. Subsequently, the impact of tandem *Eh*DH domains was tested in the oilseed plant *A. thaliana*, which is a typical self-pollinating model plant that is readily manipulated to exhibit defective forms of various metabolic functions [55–58]. The results showed that the *Eh*DH domains were functional in *A. thaliana*, as indicated by their ability to enhance seed weight, oil content, and germination rate. Furthermore, this study provides evidence that overexpression of the *Eh*DH domains of a microbial PUFA synthase has the potential to enhance seed oil content in plants.

## Conclusions

The DH domains of a PUFA synthase play key roles in double bond positioning in VLCPUFAs. Herein, the function of FabA-like DH domains derived from *E. huxleyi* were characterized by cloning and expressing them as individual proteins in a *Ts* mutant and wild-type *E. coli.* Successful complementation and functional expression of these PUFA synthase DH domains embedded in *E. coli* effectively elucidated the potential molecular mechanism of VLCPUFA biosynthesis in this species. Moreover, the expression of the tandem DH domains from *E. huxleyi* in *A. thaliana* positively impacted seed lipid accumulation, weight, and germination rate. Thus, our results provide a novel strategy to enhance oil and UFA production through the heterologous expression of a PUFA synthase domain in oilseed plants.

## Materials and methods

### Strains, plasmids, and media

*Emiliania huxleyi* was purchased from Shanghai Guangyu Biological Technology Co., Ltd. (Shanghai, China). The *E. coli* strains Trans5α Chemically Competent Cells, *E. coli* strain BL21 Star (DE3) cells were purchased from TransGen Biotech (Beijing, China), *E. coli* strain Rosetta (DE3) cells were purchased from Thermo Fisher Scientific (Waltham, MA, USA), and grown in Luria–Bertani (LB) medium (1% peptone, 0.5% yeast extract, and 1% sodium chloride). The temperature-sensitive mutant FabA (*Ts*) (IH131) was ordered from the National BioResource Project (NBRP, Japan). Expression vectors pBAD and pET32a were purchased from Invitrogen Co. (Carlsbad, CA, USA).

### Reagents

The TIANperp Mini Plasmid Kit (DP103-02), ProteinFind Anti-His Mouse Monoclonal Antibody, and ProteinFind Goat Anti-Mouse IgG(H + L) HRP Conjugate were purchased from Tiangen Biotech Co., Ltd. (Beijing, China). The Phanta Super-Fidelity DNA Polymerase and 2*Taq Master Mix (p111/p112) were purchased from Vazyme Biotech Co., Ltd (Nanjing, China). CutSmart EcoRI-HF (R3104V), CutSmart Hind III-HF (R3104V), and Q5 High-Fidelity DNA Polymerase (M0491S) were purchased from New England Biolabs Inc (Ipswich, MA, USA). The DNA purification and plasmid extraction kits were purchased from Tianyi Huiyuan Biotech Co., Ltd. (Beijing, China). The ClonExpress One-Step Cloning Kit (c112) was purchased from Vazyme. The Tks Gflex^™^ DNA Polymerase was purchased from TaKaRa (Otsu, Japan). The SuperScript^™^ III Reverse Transcriptase was purchased from Invitrogen, and the PowerUp^™^ SYBR^™^ Green Master Mix from Thermo Fisher Scientific (Waltham, MA, USA). Goldview (10,000*DMSO) and 1 kb plus DNA Marker (100–10,000 bp) were purchased from Biosharp (Beijing, China). GC-grade solvents, such as ethanol, methanol, chloroform, acetic acid, and hexane, were purchased from Biosharp. Fatty acids, including nonadecanoic acid (19:0, CAS:646-30-0), heptanoic acid (17:0), and their fatty acid methyl ester (FAME) standard mix were purchased from Rayzbio Technology Co., Ltd (Shanghai, China). All other chemicals were purchased from domestic chemical suppliers. The *E. coli* media were purchased from Biobasic Inc (ON, Canada).

### Sequence analysis of the two FabA-like DH domains of PUFA synthase from *E. huxleyi*

Sequence analysis of the PUFA synthase-like mega-enzyme from *E. huxleyi* was performed using the National Center for Biotechnology Information (NCBI) database (https://www.ncbi.nlm.nih.gov/). Simultaneously, the NCBI database was searched to identify genes similar to *Eh*DH, such as *E. coli* FabA, *Schizochytrium* PUFA-DH_1_, *Schizochytrium* PUFA-DH_2_, *Shewanella fidelis* PUFA-DH_1_, *Shewanella fidelis *PUFA-DH_2_, etc. Subsequently, a multiple sequence alignment was performed using the software package MegAlign (https://www.dnastar.com/workflows/multiple-sequence-alignment/) to identify putative domains and active sites, which were confirmed by online homology modeling tools such as SWISS-MODEL(https://swissmodel.expasy.org/), SAVES v6.0 (https://www.saves.mbi.ucla.edu/), and Phyre2 (https://www.sbg.bio.ic.ac.uk/phyre2). The 3D models of the two FabA-type DH genes were constructed with the software PyMOL to obtain more relevant structural information.

### Cloning and expression of *Eh*DH domains in *E. coli*

To clone and express the *Eh*DH_1_ and *Eh*DH_2_ in *E. coli*, the RNA was isolated from *E. huxleyi*, then reversed transcribed to cDNA. Six primers with a stop codon at the C-terminus were designed to amplify sequences from the open reading frame (ORF) of *E. huxleyi*. The DH_1_ domain was amplified using the primers F DH_1_ and R DH_1_; the DH_2_ domain was targeted by the primers F DH_2_ and R DH_1_; the tandem DH_1_-DH_2_ domain was amplified with the primers F DH_1_ and R DH_2_; the *FabA* gene from *E. coli* was amplified with the primers F-FabA and R-FabA (Additional file 1: Table S3). Amplicons with the expected size were first cloned into an intermediate pET32a vector to preserve the gene of interest [36].

For expression plasmid construction, the DH sequences from *E. huxleyi*, and *FabA* sequences from the pGEM-T vector, were sub-cloned into a His-tagged vector pBAD for complementation testing, and plasmid vector pET32a for the overexpression assay. The purified *Eh*DH and *FabA* DNA, and expression vectors were digested with restriction enzymes EcoRΙ and HindIII and ligated according to the standard procedure. The recombinant expression vectors were transformed into competent *E. coli* Trans5α cells for propagation. The recombinant plasmids were characterized by restriction enzyme digestion and sequencing.

### Site-direct mutagenesis of the DH domains

The multiple amino acid sequence alignment led to identification of specific *Eh*DH catalytic active-site residues. *Eh*DH_1_, *Eh*DH_2_, and *Eh*DH_1_-DH_2_ genes cloned in plasmid vectors were used as templates; the primers listed in Additional file 1: Table S4 were used for PCR-based site-directed mutagenesis, by which the active-site residues D or E were substituted with A. Two overlapping sequences with the mutated site and complementary region were amplified separately by PCR using mutagenic and flanking primers, respectively. Subsequently, two overlapping fragments were fused with flanking primers through overlapping PCR [59]. The DH_1_-M-UP domain from the ORF was amplified with primers F DH_1_ and DH_1_-M-UP-R, while the DH_1_-M-DOWN domain was targeted by primers R DH_1_ and DH_1_-M-DOWN-F. The DH_2_-M-UP domain from the ORF was amplified with primers F DH_2_ and DH_2_-M-UP-R, while the DH_2_-M-DOWN domain was targeted by primers R DH_2_ and DH_2_-M-DOWN-F. The DH_1_-M-DH_2_-UP domain from the ORF was amplified with primers F DH_1_ and DH_1_-M-UP-R while the DH_1_-M-DH_2_-DOWN domain was targeted by primers R DH_2_ and DH_1_-M-DOWN-F. The DH_1_DH_2_-M-UP domain from the ORF was amplified with primers F DH_1_ and DH_2_-M-UP-R, while the DH_1_DH_2_-M-DOWN domain was targeted by primers R DH_2_ and DH_2_-M-DOWN-F. (Table S4). After the cloned mutant gene fragments were recovered from the gel, the respective upstream and downstream genes were used as templates to obtain the complete target fragment overlapping PCR. The target fragment was purified by homologous recombination, ligated, transformed into Trans5α *E. coli* cells, and incubated at 37 °C for 12 h. The recombinant plasmids were confirmed by restriction digestion and DNA sequencing. The confirmed plasmids were transformed into the *E. coli* strain Rosetta for functional analysis.

### Overexpression of the DH domains in wild-type *E. coli*

Overexpression analysis was performed in the wild-type *E. coli* strain Rosetta. The expression plasmids and control vector pET32a were transformed into competent cells and selected on LB agar plates containing 100 µg/mL ampicillin. Ten colonies of the positive transformants harboring the expression plasmids were grown overnight in 500 µL of LB medium with 100 µg/mL ampicillin at 37 °C. The recombinant strains were confirmed by PCR. A 500 µL aliquot of the overnight culture was inoculated into 50 mL of fresh LB medium containing ampicillin and incubated aerobically at 37 °C until an OD_600_ of 0.4–0.6 was reached. Subsequently, 0.2 mM isopropyl β-D-1-thiogalactopyranoside (IPTG) was added to the cultured cells and incubated at 15 °C to induce expression. The fatty acid profiles of *E. coli* Rosetta transformants expressing different DH domains were analyzed by gas chromatography (GC) [32].

### Complementation of *E. coli* mutants defective in dehydratase-isomerase activity with the DH domains

Temperature-sensitive mutants of *E. coli* strain FabA (*FabA Ts*) exhibited temperature-sensitive UFA auxotrophy and were derived from the lysogenic *E. coli* strain K-12, and mutagenized by N-methyl-N′-nitrosoguanidine (NTG) [55]. This mutant grew at 37 °C, however, did not survive at 30 °C. The competent cells of the *FabA Ts* mutant were prepared according to the protocol described by Chung et al. [60]. Recombinant plasmids pBAD-*Eh*DH_1_, pBAD-*Eh*DH_2_, pBAD-*Eh*DH1DH_2_, pBAD-*Eh*DH_1_-M, pBAD-*Eh*DH_2_-M, pBAD-*Eh*DH_1_-M-DH_2_, pBAD-*Eh*DH_1_-DH_2_-M, pBAD-*Tc*DH_1_DH_2_, and control vector pBAD were transformed into FabA *Ts* mutant competent cells as described above; the transformants were spread on plates containing 100 µg/mL ampicillin and grown at 30 °C overnight. Transformant colonies were screened by PCR with a vector-specific forward primer (F-pBAD) and a gene-specific reverse primer. Positive colonies were inoculated into 2 mL of liquid LB medium with 100 μg/mL ampicillin and incubated at 30 °C overnight. For the complementation plate assay, 2 μL of cell cultures were dropped on LB agar plates containing 100 μg/mL ampicillin and 0.02% arabinose in duplicate. One plate was incubated at 30 °C, while the other was grown at 37 °C.

### Fatty acid analysis of *E. coli*

The fatty acid profiles of the cell cultures were analyzed through GC. Following inducible expression, the cells were harvested by centrifugation at 3000 × *g* for 15 min and washed twice with one-tenth volume of binding buffer (50 mM Tris-HCl pH 8.0, 100 mM NaCl, 0.1 mM EDTA, and 5 mM imidazole). An equal cell biomass (OD_600_ = 5) was used for fatty acid analysis with an internal standard of 10 µL of 10 µg/mL nonadecanoic acid (19:0) in GC-grade hexane for quantification. Total fatty acids in *E. coli* samples were converted to FAMEs by adding 2 mL of 1% sulfuric methanol to the dry sample and mixing thoroughly with a pipette. The sample was vortexed, transferred to a 10-mL glass tube with a screw cap, and heated at 80 °C for 1 h. After transmethylation, 1 mL of 0.9% NaCl and 2 mL of GC-grade hexane were added to the sample and mixed by vortexing before centrifuging at 1000 rpm for 5 min for phase separation at 25 °C. The upper hexane phase was transferred to a new glass tube. Extraction was carried out once more with 2 mL of GC-grade hexane, followed by centrifugation similar to the previous steps. Both extracted samples were combined and dried under N_2_ gas in the fume hood. Subsequently, 300 μL of GC-grade hexane was added to resolubilize the sample, which was transferred to a GC vial with an insert. The sample was subjected to the GC analysis using an Agilent 7890A system installed with a DB-5 column. The content of C14:0, C16:0, C16:1–9, C18:0, and C18:1–11 was recorded and used to calculate their specific concentrations according to the internal standard.

### Plant materials and growth conditions

Wild-type *A. thaliana* seeds were surface-sterilized and kept in the dark for 3 days at 4 °C. Seeds were germinated on a half-strength MS medium, and the plates were placed in a growth chamber set to 22 °C under a 16-h-night (120 µEm^−2^ s^−1^)/8-h-dark photoperiod. The germination rate of *A. thaliana* was documented.

### Construction of plant expression vectors

To express the DH domain in *A. thaliana*, a recombinant construct was generated using selection and screening marker genes. The binary plasmid comprising three expression cassettes was constructed to express the tandem *Eh*DH domains in *A. thaliana*. For the functional analysis of the domain effects on fatty acid biosynthesis and oil production in *A. thaliana*, the construct expressing this domain under the 35S promoter was introduced into wild-type *A. thaliana*. The plastidial overexpression construct contained a tandem *Eh*DH domain from the PUFA-like synthase (Accession: XP_005774327.1) fused with a functional chloroplast transit peptide (CTP) at the N-terminus and an eGFP from *A. victoria* encoding a modified GFP, each under the control of a seed-specific napin promoter for screening transgenic seeds, as well as an antibiotic kanamycin-resistance gene under the control of a constitutive NOS promoter.

### Transcriptional expression analysis

The seeds of three transgenic lines (OE-4, OE-5, OE-11) and wild-type *A. thaliana* were collected and frozen in liquid N_2_. RNA was extracted using the Qiagen RNeasy Mini Kit and treated with DNase I for 30 min to digest contaminating DNA. Biosynthesis of complementary DNA was carried out using the SuperScript^™^ III Reverse Transcriptase, and quantitative RT-PCR was performed using PowerUp^™^ SYBR^™^ Green Master Mix. The PCR conditions were as follows: 50 °C for 2 min, 95 °C for 2 min, 40 cycles of 95 °C for 5 s, 60 °C for 1 min, and one cycle of 95 °C for 15 s, 60 °C for 1 min, and 95 °C for 15 s for the melting curve stage. The expression of the housekeeping gene *At*Actin-2 (AT3G18780) was used as an internal reference. The expression of the *Eh*DH_1_-DH_2_ domain and *At*Actin-2 in the developing siliques of the *Eh*DH_1_-DH_2_/WT *A. thaliana* plants was analyzed by semi-quantitative RT-PCR. The expression of the *Eh*DH_1_-DH_2_ domain in the developing siliques of the *Eh*DH1-DH2/WT plants was analyzed by quantitative RT-PCR.

### Fatty acid composition and oil content analysis

The fatty acid profiles of the seeds were analyzed via GC analysis. A single seed was used for fatty acid analysis with an internal standard of 5 µL of 10 µg/mL heptanoic acid (17:0) for quantification. The samples were converted to FAME samples by adding 2 mL of 1% sulphuric acid in methanol to the dry sample. The FAME samples were vortexed and transferred to a 10 mL glass tube with a screw cap and heated at 80 °C for 1 h. After transmethylation, the sample was mixed with 2 mL of hexane and 1 mL of 0.9% NaCl and vortexed before centrifuging at 1000 rpm for 5 min for phase separation at room temperature. The upper hexane phase was transferred to a new glass tube. The extraction process was carried out once more with 2 mL of hexane and centrifuged similar to the previous steps. Both extracted samples were combined and dried under N_2_ gas in a fume hood. Subsequently, 300 μL of GC-grade hexane was added to resolubilize the sample and transferred to a GC vial with an insert. The samples were subjected to GC analysis using an Agilent 7890A system installed with a DB-5 column.

## Supplementary Information

**Additional file 1: Table S1.** Production of SFAs and UFAs in wild type *Escherichia coli* Rosetta overexpressing *Eh*DH domains. **Table S2.** Production of SFAs and UFAs in wild-type *Escherichia coli* Rosetta overexpressing the mutated *Eh*DH domains. **Table S3.** Primers used for DH domain amplifications. **Table S4.** Primers used for site-directed mutagenesis of DH domains. **Table S5.** Primers used for plant experiment. **Figure S1.** Expression of recombinant proteins when induced with 0.2 mM IPTG in *Escherichia coli* (*E. coli*). a SDS-PAGE and WB analysis of the extracellular secretion from *E. coli* Rosetta strains overexpressing EhDH domains. b WB analysis of the extracellular secretion from *E. coli* Rosetta strains expressing site-directed mutagenized EhDH domains. SDS-PAGE, the sodium dodecyl sulphate-polyacrylamide gel electrophoresis; WB, western blotting. The marker used in this experiment is 180 kDa Prestained Protein Marker (180, 130, 100, 70, 55, 40, 35, 25, 15 kDa); 1, pET32a-*Eh*DH_1_; 2, pET32a-*Eh*DH_1_-M; 3, pET32a-*Eh*DH_2_; 4, pET32a-*Eh*DH_2_-M; 5, pET32a-*Eh*DH_1_-DH_2_; 6, pET32a-*Eh*DH_1_-M-DH_2_; 7, pET32a-*Eh*DH_1_-DH_2_-M; 8 pET32a-*Tc*DH_1_-DH_2_; 9 pET32a (negative control). **Figure S2.** Schematic representation of the T-DNA region of the binary plasmid vector expressing tandem EhDH domains.

## Data Availability

All data generated or analyzed during this study are included in this article and its additional files.
