# Supplementary material for: Functional analysis of the dehydratase domains of the PUFA synthase from Emiliania huxleyi in Escherichia coli and Arabidopsis thaliana
Source: Biotechnol Biofuels Bioprod. 2022 Nov 15;15:123. doi: 10.1186/s13068-022-02223-w (PMC9667614; doi:10.1186/s13068-022-02223-w)
Supplement: Supplementary file 1 — Additional file 1: Table S1. Production of SFAs and UFAs in wild type Escherichia coli Rosetta overexpressing EhDH domains. Table S2. Production of SFAs and UFAs in wild-type Escherichia coli Rosetta overexpressing the mutated EhDH domains. Table S3. Primers used for DH domain amplifications. Table S4. Primers used for site-directed mutagenesis of DH domains. Table S5. Primers used for plant experiment. Figure S1. Expression of recombinant proteins when induced with 0.2 mM IPTG in Escherichia coli (E. coli). a SDS-PAGE and WB analysis of the extracellular secretion from E. coli Rosetta strains overexpressing EhDH domains. b WB analysis of the extracellular secretion from E. coli Rosetta strains expressing site-directed mutagenized EhDH domains. SDS-PAGE, the sodium dodecyl sulphate-polyacrylamide gel electrophoresis; WB, western blotting. The marker used in this experiment is 180 kDa Prestained Protein Marker (180, 130, 100, 70, 55, 40, 35, 25, 15 kDa); 1, pET32a-EhDH1; 2, pET32a-EhDH1-M; 3, pET32a-EhDH2; 4, pET32a-EhDH2-M; 5, pET32a-EhDH1-DH2; 6, pET32a-EhDH1-M-DH2; 7, pET32a-EhDH1-DH2-M; 8 pET32a-TcDH1-DH2; 9 pET32a (negative control). Figure S2. Schematic representation of the T-DNA region of the binary plasmid vector expressing tandem EhDH domains. [file 13068_2022_2223_MOESM1_ESM.docx]

Submitted to **Biotechnology for Biofuels and Bioproducts**

**Manuscript title:** Functional analysis of the dehydratase domains of the PUFA synthase from *Emiliania huxleyi* in *Escherichia coli* and *Arabidopsis thaliana.*

**Authors:** Bihan Chen^1,2^, Feng Wang^1,2^, Xi Xie^1,2*^, Huifan Liu^1,2^, Dongjie Liu^1,2^, Lukai Ma^1,2^, Gengsheng Xiao^1,2^, Qin Wang^1,2^

**The affiliation** **and address:**

^1^ Guangdong Provincial Key Laboratory of Lingnan Specialty Food Science and Technology, Guangzhou, China

^2^ College of Light Industry and Food, Zhongkai University of Agriculture and Engineering, Guangzhou, China

* Corresponding author: Xi Xie

Guangdong Provincial Key Laboratory of Lingnan Specialty Food Science and Technology

**E-mail address, telephone and fax number:**

College of Light Industry and Food

Zhongkai University of Agriculture and Engineering

Guangzhou, China

Phone number: +8618126708703

Fax: +86 (0758)2220421

Email address: [xixie31@hotmail.com](mailto:xixie31@hotmail.com)

**Table S1** Production of SFAs and UFAs in wild type *Escherichia coli* Rosetta overexpressing *Eh*DH domains.

| **Strain** | **Amount of fatty acids produced (mg/L)** | | | **UFA/SFA ratio** |
| --- | --- | --- | --- | --- |
|  | **Total** | **UFA** | **SFA** |  |
| EV | 126.79±3.38^C^ | 76.86±2.07^C^ | 49.93±1.32^C^ | 1.54 |
| *Eh*DH_1_ | 181.92±2.94^B^ | 94.73±1.96^B^ | 87.19±1.74^A^ | 1.09 |
| *Eh*DH_2_ | 168.51±3.00^B^ | 94.66±2.29^B^ | 73.85±1.15^B^ | 1.28 |
| *Eh*DH_1_-DH_2_ | 181.99±0.24^B^ | 93.98±0.79^B^ | 87.72±1.03^A^ | 1.07 |
| *Tc*DH_1_-DH_2_ | 220.36±10.55^A^ | 130.17±7.29^A^ | 90.20±4.98^A^ | 1.45 |

EV, *E. coli* Rosetta with an empty vector; *Eh*DH_1_, *E. coli* Rosetta overexpressing the *Eh*DH_1_ domain; *Eh*DH_2_, *E. coli* Rosetta overexpressing the *Eh*DH_2_ domain; *Eh*DH_1_-DH_2_, *E. coli* Rosetta overexpressing the *Eh*DH_1_ and *Eh*DH_2_ domains; *Tc*DH_1_-DH_2_, *E. coli* Rosetta overexpressing the *Tc*DH_1_ and *Tc*DH_2_ domains. Fatty acid abbreviations: SFAs, saturated fatty acids; UFAs, unsaturated fatty acids; Total, total fatty acids. Values are reported as the means ± standard deviations for three independent biological replicates. The means with the same letters are not statistically significantly different. Statistical analysis of the results was conducted using one-way analysis of variance (*P* < 0.05).

**Table S2** Production of SFAs and UFAs in wild-type *Escherichia coli* Rosetta overexpressing the mutated *Eh*DH domains.

| **Strain** | **The amount of fatty acids produced (mg/L)** | | | **UFA/SFA ratio** |
| --- | --- | --- | --- | --- |
|  | **Total** | **UFA** | **SFA** |  |
| EV | 126.79±3.38^D^ | 76.86±2.07^B^ | 49.93±1.32^B^ | 1.54 |
| *Eh*DH_1_-M | 159.71±6.13^B^ | 89.96±12.91^B^ | 51.86±7.43^B^ | 1.72 |
| *Eh*DH_2_-M | 148.94±1.59^C^ | 94.03±1.33^B^ | 54.91±0.27^B^ | 1.71 |
| *Eh*DH_1_-M-DH_2_ | 198.32±2.11^A^ | 128.32±1.87^A^ | 70.00±0.32^A^ | 1.83 |
| *Eh*DH_1_-DH_2_-M | 204.58±3.66^A^ | 129.67±1.37^A^ | 74.91±2.30^A^ | 1.73 |

EV, *E. coli* Rosetta with an empty vector; *Eh*DH_1_-M, *E. coli* Rosetta overexpressing the mutated *Eh*DH_1_ domain; *Eh*DH_2_-M, *E. coli* Rosetta overexpressing the mutated *Eh*DH_2_ domain; *Eh*DH_1_-M-DH_2_, *E. coli* Rosetta overexpressing mutated DH_1_ along with DH_2_; *Eh*DH_1_-DH_2_-M, *E. coli* Rosetta overexpressing mutated DH_2_ along with DH_1_. Fatty acid abbreviations: SFAs, saturated fatty acids; UFAs, unsaturated fatty acids; Total, total fatty acids.

Values are reported as the means ± standard deviations of three independent biological replicates. The means with the same letters are not statistically significantly different. Statistical analysis of the results was conducted using one-way analysis of variance (*P* < 0.05).

**Table S3** Primers used for DH domain amplifications.

| **Name** | **Sequence(5′→3′)** | **Annotation** |
| --- | --- | --- |
| F-*Eh*-DH_1_ | TATCGGATCCGAATTCATGGATGAACGTCTGCTGC | EcoRⅠ site (underlined) |
| R-*Eh*-DH_1_ | GTGCGGCCGCAAGCTTTACTGCAGGGCAACACC | HindⅢ site (underlined) |
| F-*Eh*-DH_2_ | TATCGGATCCGAATTCATGGAAGGTCCGCCTACCG | EcoRⅠ site (underlined) |
| R-*Eh*-DH_2_ | GTGCGGCCGCAAGCTTTACTGCACCGCACCAACAC | HindⅢ site (underlined) |
| F-FabA | GAATTCATTCCGGGGATCCGTCG | EcoRI site (underlined) |
| R-FabA | AAGCTTGAAGCAGCTCCAGCCTAC | HindIII site (underlined) |

**Table S4** Primers used for site-directed mutagenesis of DH domains.

| **Name** | **Sequence(5′→3′)** | **Annotation** |
| --- | --- | --- |
| R-*Eh*-DH_1_-M-UP | CTGATCGTTTTTGAATGCACACGGGAAATACCA | Mutation site (underlined) |
| F-*Eh*-DH_1_-M-DOWN | TGGTATTTCCCGTGTGCATTCAAAAACGATCAG | Mutation site (underlined) |
| R-*Eh*-DH_2_-M-UP | CGGGTCACACCAGAATGCACAAGAGAAAAACCA | Mutation site (underlined) |
| F-*Eh*-DH_2_-M-DOWN | TGGTTTTTCTCTTGTGCATTCTGGTGTGACCCG | Mutation site (underlined) |
| R-*Eh*-DH_1_-M-UP | CTGATCGTTTTTGAATGCACACGGGAAATACCA | Mutation site (underlined) |
| F-*Eh*-DH_1_-M-DOWN | TGGTATTTCCCGTGTGCATTCAAAAACGATCAG | Mutation site (underlined) |
| R-*Eh*-DH_2_-M-UP | CGGGTCACACCAGAATGCACAAGAGAAAAACCA | Mutation site (underlined) |
| F-*Eh*-DH_2_-M-DOWN | TGGTTTTTCTCTTGTGCATTCTGGTGTGACCCG | Mutation site (underlined) |

**Table S5** Primers used for plant experiment.

| **Category** | **Name** | **Sequence (5’ to 3’)** | **Annotation** |
| --- | --- | --- | --- |
| **Expression construct preparation** | F-CTP | ATGGCTTCCTCTATGCTCTCTTCCG | Primers for cloning CTPs |
|  | R-CTP | GAATTCCTTGTCGTCGTCGTCCTTGTAGTC |  |
|  | F-*Eh*DH-P | GCGAATTCATGGCGCTCCGCGTCA | Primers for cloning *Eh*DH_1_-DH_2_ domain |
|  | R*-Eh*DH-P | GCAAGCTTCTAGAAGGAGCGGTCGCCGA |  |
| **qRT-PCR** | F-Actin2 | GATTCCTGGACCTGCCTCAT | Actin-2 primers for qPCR |
|  | R-Actin2 | TACCCGATGGGCAAGTCA |  |
|  | DH-qPCR-F | GGGTGAAGGTTGCGTTAG | *Eh*DH_1_-DH_2_ primers for qPCR |
|  | DH-qPCR-R | ACGAATACGCAGACCAGTT |  |

**Fig. S1**


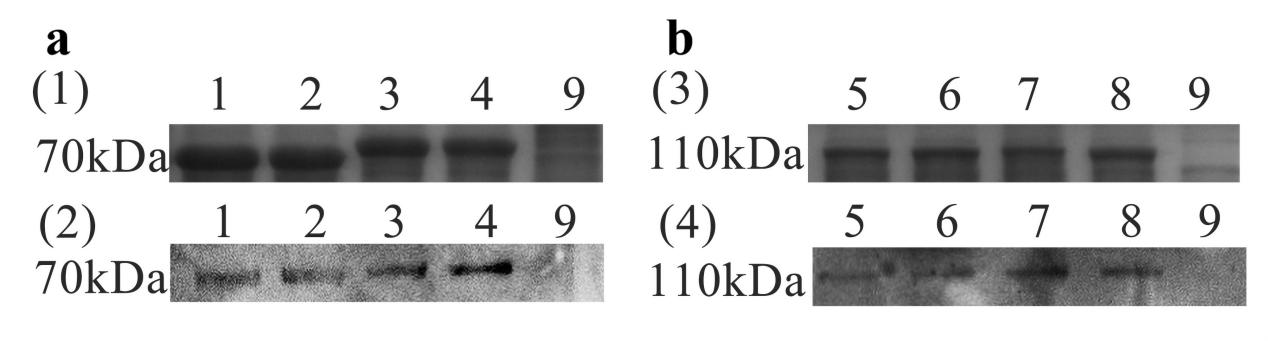


**Fig. S1** Expression of recombinant proteins when induced with 0.2 mM IPTG in *Escherichia coli* (*E. coli*). **a** SDS-PAGE and WB analysis of the extracellular secretion from *E. coli* Rosetta strains overexpressing *Eh*DH domains. **b** WB analysis of the extracellular secretion from *E. coli* Rosetta strains expressing site-directed mutagenized *Eh*DH domains. SDS-PAGE, the sodium dodecyl sulphate-polyacrylamide gel electrophoresis; WB, western blotting. The marker used in this experiment is 180 kDa Prestained Protein Marker (180, 130, 100, 70, 55, 40, 35, 25, 15 kDa); 1, pET32a-*Eh*DH_1_; 2, pET32a-*Eh*DH_1_-M; 3, pET32a-*Eh*DH_2_; 4, pET32a-*Eh*DH_2_-M; 5, pET32a-*Eh*DH_1_-DH_2_; 6, pET32a-*Eh*DH_1_-M-DH_2_; 7, pET32a-*Eh*DH_1_-DH_2_-M; 8 pET32a-*Tc*DH_1_-DH_2_; 9 pET32a (negative control).

**Fig. S2**


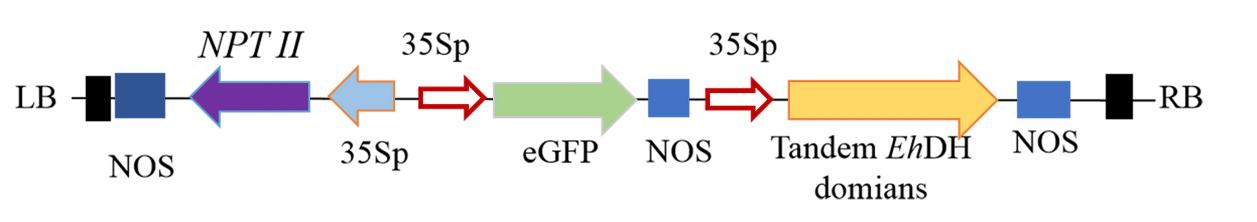


**Fig. S2** Schematic representation of the T-DNA region of the binary plasmid vector expressing tandem *Eh*DH domains.
